# The effect of age on third molar extraction difficulty: a retrospective cross-sectional cohort study

**DOI:** 10.2340/aos.v85.45308

**Published:** 2026-01-20

**Authors:** Abiel Noro, Johanna Snäll, Irja Ventä

**Affiliations:** a Department of Oral and Maxillofacial Diseases, Faculty of Medicine, University of Helsinki, Helsinki, Finland; b Department of Oral and Maxillofacial Diseases, University of Helsinki and Helsinki University Hospital, Helsinki, Finland

**Keywords:** Molar, third, tooth extraction, primary health care, age group, surgery, oral

## Abstract

**Objective:**

This study aimed to clarify whether third molar extraction becomes more difficult as the patient ages. Previous research results on the topic are contradictory.

**Materials and methods:**

All 12,649 third molar extractions in Helsinki’s primary care during 2016 were retrieved from electronic records using treatment codes. The primary outcome was extraction difficulty, classified as simple (forceps) or surgical (raising a flap). The secondary outcome was an extraction labelled as demanding. Predictor variables were age, sex, and the jaw (maxilla/mandible). Statistical analyses included χ^2^ test and binomial logistic regression.

**Results:**

Mean age of the patients was 32.2 years (range 12–97 years). Simple extractions in the maxilla (*P* < 0.001) and all surgical extractions (*P* < 0.001) were more likely to be demanding at a higher age. In the mandible, simple extractions were easier at a higher age (odds ratio [OR] 0.971; 95% confidence interval [CI] 0.964; 0.978). Surgical extraction was 1.4 times (95% CI 1.145; 1.766) more likely to be demanding in men and twice (95% CI 1.143; 3.462) as demanding in the mandible.

**Conclusions:**

Surgical and maxillary simple extractions were more difficult at a higher age. Surprisingly, in the mandible, simple extractions were easier at a higher age.

## Introduction

Higher age of the patient in third molar surgery is associated with a higher rate of postoperative complications such as alveolitis, persistent pain, or paresthesia [1–4]. This is also reported in extractions performed under local anesthesia [5]. The complication rate depends on several patient-specific, radiological, and operative factors, including surgical difficulty [6]. However, the extraction method among younger and older patients has not been comprehensively examined.

In a British study on simple and surgical extractions of third molars performed under general anesthesia, higher age of the patient predicted significantly higher difficulty of extractions [7]. Similarly, in studies from Iceland and Nigeria, higher age was associated with technically more difficult extractions [8, 9]. In those studies of mandibular third molar extractions, the difficulty of extraction was assessed by radiograph features and procedure duration.

A completely opposite conclusion was reached in a US study, where third molar extractions of adolescents were recorded with treatment codes indicating more demanding procedures than those of older patients [10]. In their study on insured patients, almost all third molar extractions in patients aged 16 years and younger are recorded with procedure codes of partial or full bony impactions. The mean age of their patients was 18 years; this is notably lower than in many other third molar studies, which report mean ages of 25.2–36.4 years [1, 7, 9, 11, 12].

Another US study on extracted third molars reported a similar relationship between patient age and degree of impaction of extracted third molars [13]. Extractions of patients younger than 21 years comprised partial or complete bony impacted third molars in 77% of cases, whereas among patients older than 30 years more than half of extracted third molars were fully erupted. Thus, previous literature yields variable findings regarding the association between a patient’s age and difficulty level of third molar extraction. Therefore, it is appropriate to broaden this topic using a wide age range of patients from primary care, covering simple and surgical extractions from both jaws.

The purpose of this study was to examine the association of the patient’s age with the level of difficulty of third molar extraction. The hypothesis was that third molar extractions are more challenging among older patients.

## Materials and methods

### Study design

A retrospective cross-sectional cohort study on third molar extractions was designed and performed on patients treated at the Department of Social Services and Health Care of the City of Helsinki, Finland. This primary care unit serves altogether 1.6 million people from the capital area and surrounding regions by offering oral health care services to residents of Helsinki and emergency dental services in the evenings and on weekends also to the neighboring cities. In Finland, public dental health care is free for patients under 18 years of age. The staff of the unit comprises approximately 200 dentists, 10 of whom are specialized in oral surgery. Most extractions (93%) of third molars in this unit are performed under local anesthesia.

Patient data of all extracted third molars over a 1-year period (2016) were retrieved from the electronic patient register according to treatment code (Table 1). Extractions done in private clinics or hospitals do not appear in this register. Reporting in the study follows the Strengthening the Reporting of Observational Studies in Epidemiology (STROBE) guidelines.

**Table 1:** Treatment codes displayed in the classification of the Finnish Institute for Health and Welfare (THL) [14] for tooth extraction used in the data search.

Treatment code	Operation	Definition
**Simple extractions**		
EBA00	Simple extraction	With elevator and forceps
EBA05	*Demanding* simple extraction	Separating a tooth without raising a flap
EBA30	Extraction of root	Extraction of a root with elevator and forceps
**Surgical extractions**		
EBA10	Surgical extraction	Raising a flap, followed mostly by osteotomy and separation of tooth
EBA12	*Demanding* surgical extraction	Deep and difficult impactions
**Other extraction procedures**		
EBA15	Extraction of several teeth	Extraction of at least four teeth from a jaw when eradicating an infection
EBA20	Hemisection	Raising a flap, separation, and extraction part of a tooth
EBA99	Other surgical extraction	Other extraction of tooth

### Study variables

The following variables were recorded from the data: age and sex of patient, identification of tooth and jaw, and treatment code indicating method of extraction.

The primary outcome variable was the difficulty level of third molar extraction. It was determined according to the treatment code and categorized as simple extraction (with elevator and forceps) and surgical extraction (including flap raising with osteotomy and sectioning when necessary) (Table 1). The treatment codes under the heading of other extraction procedures in Table 1 were excluded from the analysis because their degree of difficulty was unclear (*n* = 79 procedures).

The secondary outcome variable was an extraction labelled additionally as demanding or not. This label was attached to two treatment codes: demanding simple extraction and demanding surgical extraction (Table 1). Predictor variables were age, sex, and jaw (maxilla or mandible).

### Ethical considerations

This study followed the 1964 Declaration of Helsinki on medical research protocols and ethics [15]. The Department of Social Services and Health Care of the City of Helsinki approved the secondary utilization of this healthcare data (HEL 2023-014505). This study was register-based and retrospective without any patient interventions, and thus, additional institutional ethics committee approval was unnecessary. Based on the General Data Protection Regulation (GDPR) of the European Parliament concerning personal data, results were not presented if the frequencies were less than 5, and therefore, in the analysis, some combinations of the oldest age groups were made.

### Statistical analysis

The unit of the analysis was the third molar. The age of the patient was grouped as 10−19, 20−29, 30−39, 40−49, 50−59, 60−69, 70–79, and 80 years and older. Descriptive analysis included rates of extractions cross-tabulated according to age groups. The χ^2^ test was used to analyze differences in frequencies between categorical variables. The level of significance was set at *P*-values < 0.05. SPSS Statistics version 29 (IBM Corporation, Armonk, NY, USA) was used to perform all analyses.

A binomial logistic regression analysis was performed to determine the effects of age, sex, and jaw on the likelihood of extraction being demanding. Such analyses were performed for simple and surgical extractions separately. Odds ratios (ORs) and their 95% confidence intervals (CIs) were reported. Hosmer and Lemeshow’s test was used to evaluate the model’s goodness of fit. In the preliminary analysis of simple extractions, Hosmer and Lemeshow’s test value was poor, and therefore, regression analysis of simple extractions was performed separately for the maxilla and the mandible.

## Results

The number of extracted third molars included in the study was 12,649 (49% in maxilla, 51% in mandible). These extractions were performed at 10,822 appointments; 45% of extractions were for men and 55% for women (Table 2). Patients’ mean age was 32.2 years (standard deviation [SD] 12.1; median 28.0; range 12–97 years). Oral surgeons performed 16% of third molar extractions, while general dentists carried out the remaining 84%.

**Table 2:** Description of patients attending 10,822 visits according to age group and sex.

Age group	Men		Women		Total	
(years)	*n*	%	*n*	%	*n*	%
10–19	138	3	213	4	351	3
20–29	2,161	45	3,493	58	5,654	52
30–39	1,404	29	1,398	23	2,802	26
40–49	490	10	399	7	889	8
50–59	352	7	247	4	599	6
60–69	188	4	150	2	338	3
70–79	71	1	73	1	144	1
≥ 80	22	1	23	1	45	1
Total	4,826	45	5,996	55	10,822	100

**Figure 1:**
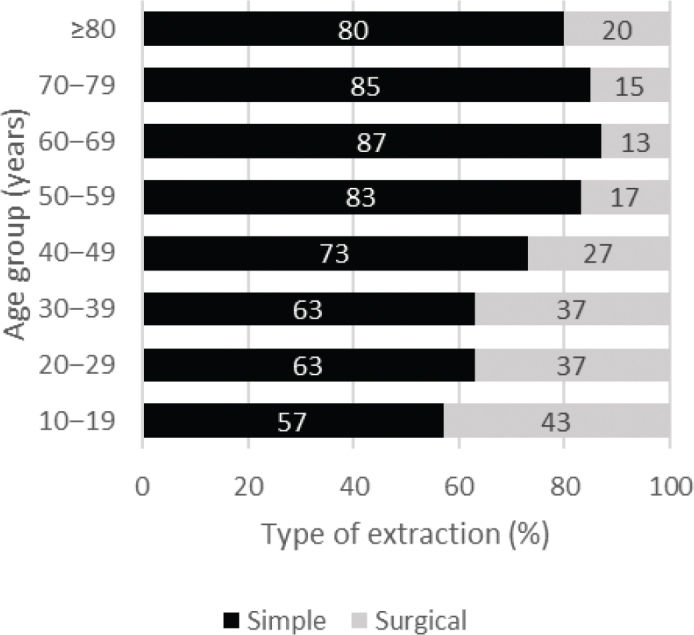
Distribution of 12,649 simple and surgical third molar extractions by age group.

When the frequencies of all extractions were analyzed together, simple extractions with forceps were more prevalent than surgical extractions in all age groups (Figure 1). This difference was most prominent in the oldest age groups. The rate of surgical extractions was reduced by half between the youngest and oldest age groups. Differences between subgroups were significant (χ^2^ = 246.85; df = 7; *P* < 0.001).

**Figure 2:**
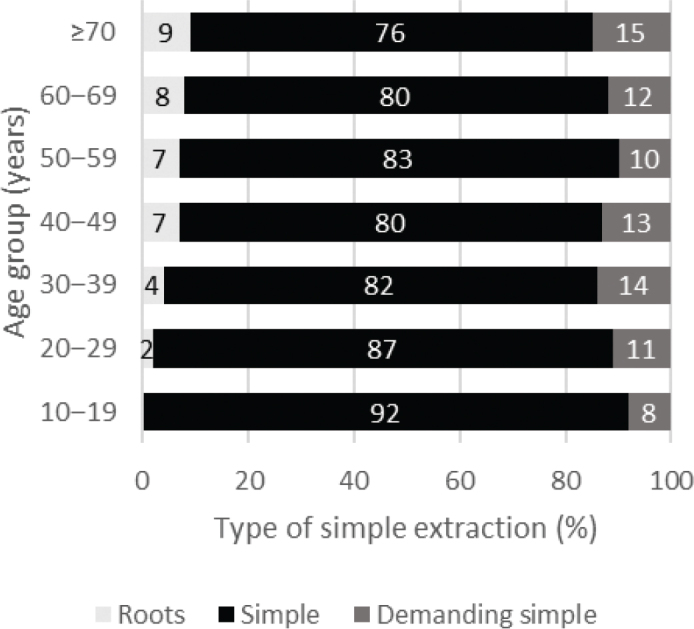
Distribution of difficulty level of 8,280 simple extractions of third molars by age group.

Regarding simple extractions, Figure 2 shows that demanding extractions in the highest age group were twice as common as in the youngest group. Differences in the difficulty level of simple extraction between age groups were significant (χ^2^ = 153.80; df = 12; *P* < 0.001).

**Figure 3:**
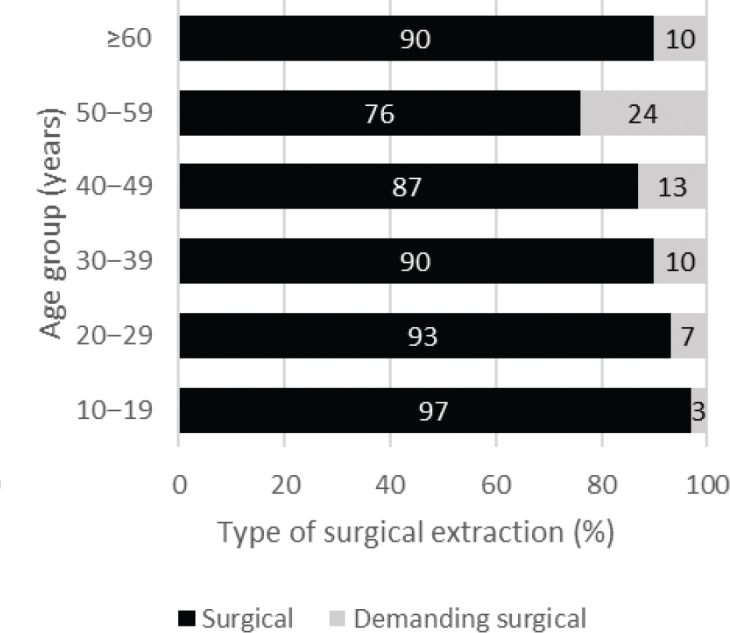
Distribution of degree of difficulty of 4,369 surgical extractions of third molars by age group.

Regarding surgical extractions, Figure 3 shows that demanding extractions were more common in the older age groups. Especially at the age of 50–59 years, demanding surgical extractions were eight times more common than in the youngest age group. Differences in the difficulty level of surgical extractions between subgroups were also significant (χ^2^ = 60.50; df = 5; *P* < 0.001).

**Figure 4:**
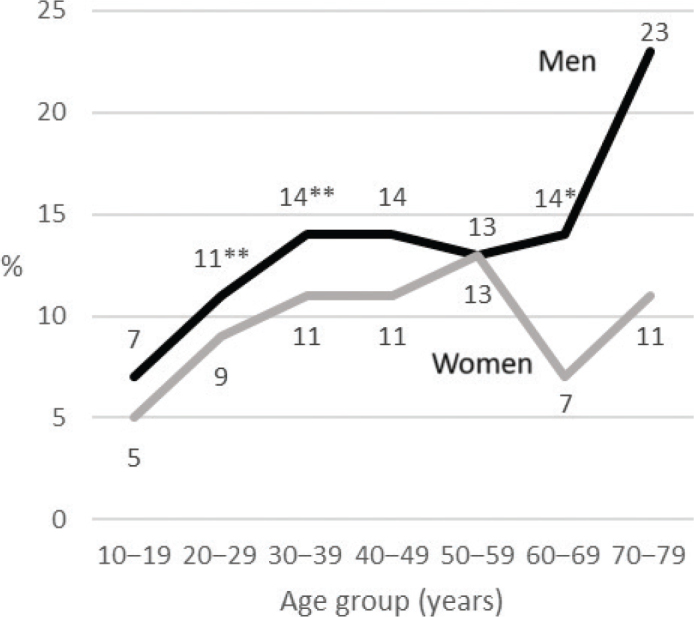
Rates of demanding extractions of all third molars in each age group according to sex. Significant differences between men and women in age groups are shown with asterisks, * *P* < 0.05 and ** *P* < 0.001.

When examining all third molar extractions together, the proportion of demanding extractions was higher in men than in women (13% vs. 9%; χ^2^ = 37.84; df = 1; *P* < 0.001). In a detailed analysis by age group, significant differences between men and women were found in three age groups (Figure 4).

In the logistic regression analysis of simple extractions in the maxilla, extractions with forceps were more likely to be demanding in older patients (Table 3). Also, in the maxilla, extraction in men was 1.4 times (95% CI 1.14; 1.81) more likely to be demanding than in women. In the mandible, the OR for the patient’s age was < 1, indicating that simple extractions with forceps were easier in older patients than in younger patients. Sex was not a significant covariate in the mandible.

**Table 3:** Logistic regression based on simple extractions in the maxilla (5,963 teeth) and the mandible (2,317 teeth) and predicting the likelihood of a third molar removal being demanding.

Jaw	Characteristic		*P*-value	OR	95% CI
Maxilla	Age		< 0.001	1.017	1.01; 1.03
	Sex				
		[Women]			
		Men	0.002	1.432	1.14; 1.81
					
Mandible	Age		< 0.001	0.971	0.964; 0.978
	Sex				
		[Women]			
		Men	0.167	1.137	0.948; 1.365

Reference group in square brackets. OR: odds ratio; CI: confidence interval. Hosmer and Lemeshow test value for the maxilla: χ^2^ = 14.02; df = 8; *P* = 0.081 and for the mandible χ^2^ = 12.29; df = 8; *P* = 0.139 (both adequate).

In the logistic regression analysis of surgical extractions (7% in maxilla, 93% in mandible), higher age rendered the procedure more demanding (OR 1.028; 95% CI 1.018; 1.037) (Table 4). Surgical extraction was 1.4 times (95% CI 1.145; 1.766) more likely to be demanding in men than in women and twice (95% CI 1.143; 3.462) as likely to be demanding in the mandible than in the maxilla.

**Table 4:** Logistic regression based on surgical extractions of 4,369 third molars and predicting the likelihood of a third molar removal being demanding.

Characteristic	*P*-value	OR	95% CI
Age	< 0.001	1.028	1.018; 1.037
Sex			
[Women]			
Men	0.001	1.422	1.145; 1.766
Jaw			
[Maxilla]			
Mandible	0.015	1.989	1.143; 3.462

Reference group in square brackets. OR: odds ratio; CI: confidence interval. Hosmer and Lemeshow test value for the model: χ^2^ = 8.13; df = 8; *P* = 0.421 (good).

## Discussion

This study aimed to clarify conflicting findings presented earlier on patient age and extraction difficulty of third molars. The main findings were that surgical extractions are more demanding in older patients than in younger ones, as were simple extractions in the maxilla. Surprisingly, simple extractions in the mandible were easier in older patients than in younger ones. The hypothesis was confirmed for surgical extractions but not for all simple extractions.

Surgical extractions were more demanding at a higher age than at a younger age. Earlier results of mandibular extractions are in concordance with our finding [7–9]. Demanding tooth extractions occurred most frequently in 50−59-year-old patients. This finding may be explained by delayed extractions. Patients and clinicians might have postponed the extraction of asymptomatic, disease-free, and deeply impacted third molars with a risk of, for example, nerve injury until the extraction became urgent. A systematic review [16] suggests that retained third molars, especially those partially erupted in the mandible, eventually become pathological over time. While deeply impacted third molars are less likely to become diseased, they may be exposed later due to bone resorption caused by adjacent tooth loss or periodontitis [17]. Late extraction may also be indicated due to cysts or tumors, although they are rare, affecting only 2% of third molars in older persons [18].

The difficulty level of simple extractions in the maxilla and the mandible differed by age. In the maxilla, simple extractions were more demanding at a higher age. In the mandible, the opposite was true; simple extractions were more demanding in younger patients. However, the level of difficulty of simple extractions has rarely been examined [19], hindering comparison with earlier studies. General reasons among older patients for demanding extractions include reduced periodontal ligament space, decreased bone flexibility, hypercementosis, and ankylosis [20–24]. In the maxilla, older patients are also at greater risk of maxillary sinus perforation and fractures of the maxillary tuberosity [25, 26]. Extraction of a deeply carious tooth in the maxilla may become difficult, which may also play a role, as caries is the main indication in older patients [1, 12].

An interesting finding of this study was that simple mandibular extractions were less demanding in older patients than in younger ones. The opp`osite could be assumed due to the anatomy of the mandible and third molars. This finding may be explained by the fact that a common reason for the extraction in older patients is periodontitis [1, 12]. In addition, periodontal pathologies of third molars in older people have been shown to be more common in the mandible than in the maxilla [18].

The rate of demanding extractions of all third molar extractions was higher in men than in women. This finding applied specifically to simple extractions in the maxilla and all surgical extractions. Previous studies on the subject have yielded conflicting results, although simple extractions have not been widely investigated in this respect. A US study [2] reported that surgical extractions were slightly more demanding and time-consuming in men than in women, while other studies have not found a significant difference between the sexes [7, 9, 27–29]. An explanation for more demanding extractions in men may be their higher bone density [30].

In this study, age, sex, and jaw were significant predictors of extraction difficulty, but other factors are also reported in the literature for surgical extractions. Anatomical variables, such as third molar angulation, type of impaction, location, root morphology, inferior alveolar nerve proximity, and bone density, as well as procedure- and clinician-related factors, including operator experience, extraction method, anesthesia type, and number of teeth extracted, also influence the difficulty [3, 7, 31, 32]. Additional factors, such as mouth opening, cheek flexibility, and even body mass index (BMI), have also been reported to influence tooth removal [7, 9]. However, according to a review article on the difficulty of surgical third molar extractions [33], the only significant independent non-radiological variables were patient’s age, operator experience, procedure type, and number of teeth extracted.

The treatment codes and their precise definition used in this study are based on the procedure classification maintained by the Finnish Institute for Health and Welfare (THL). This classification is based on the Nordic procedural classification system, known as the Nordic Medico-Statistical Committee’s (NOMESCO) procedure codes [34]. NOMESCO is used across the Nordic countries (Finland, Sweden, Norway, Denmark, and Iceland) for statistical and administrative purposes. The procedure classification has been in use in Finland since 2004, and the code sets are updated annually. In this classification, there is no specific code for coronectomy. It is possible that coronectomies are recorded under the codes for other surgical removals.

In this study, the difficulty of extraction was assessed using treatment codes classified by the THL (Table 1). Other scales for extraction difficulty level exist as well but comparing them is challenging. For example, the Modified Parant Scale [35] also includes four levels of difficulty but arranges them differently: Easy I (simple extraction requiring forceps only), Easy II (extraction requiring osteotomy), Difficult III (extraction requiring coronal sectioning), Difficult IV (complex extraction requiring radicular sectioning). However, the present approach enabled analysis of huge datasets from the electronic patient register. Most earlier studies have measured extraction difficulty using radiographs and operation duration [7–9, 24]. However, to gain a comprehensive picture of all third molar treatments, the use of treatment codes from the electronic patient register is recommended.

The findings of the study can be generalized to dental health care in all countries. However, the use of treatment codes is not universal. The strength of the study was its inclusion of both simple and surgical third molar extractions across a wide age range from both jaws, and it was not restricted to general dentists. The retrospective aspect and using treatment codes to assess the difficulty of extraction were also limitations of the study, as there was no information available on the operator’s experience over the years, the duration of the procedure, or access to radiographic images – all of which would have been useful for evaluating extraction difficulty. Specifically, the absence of radiographic data is a major limitation of this study. Using a standardized radiographic classification (e.g. Pell & Gregory or Winter [36, 37]) to assess extraction difficulty would have enhanced the study’s objectivity and reproducibility. Previous studies have examined this topic using these variables; however, in this study, big data were analyzed from a different perspective.

Another limitation was that dentists’ salaries in Finland are minimally based on these codes, which may have potentially influenced their use. Indications of such economic impact have been found in previous studies from the USA and Finland [10, 38]. Further research is needed to explore the underlying causes in relation to differences between patient age and extraction difficulty in the maxilla and mandible.

## Conclusion

This study hypothesized that third molar extractions are more complex in older patients than in younger ones. The hypothesis was confirmed for surgical extractions and simple extractions in the maxilla, but not for simple extractions in the mandible.

## Declarations

### Confirmation

All authors have reviewed and agreed to the contents of this manuscript.

### Data availability

No datasets were generated for public use during the study.

### Geolocation information

Helsinki, Finland.
